# MicroRNA-674-5p induced by HIF-1α targets XBP-1 in intestinal epithelial cell injury during endotoxemia

**DOI:** 10.1038/s41420-020-0280-5

**Published:** 2020-06-04

**Authors:** Zhihao Liu, Jie Jiang, Weigang Dai, Hongyan Wei, Xiaofei Zhang, Zhen Yang, Yan Xiong

**Affiliations:** 1 Division of Emergency Medicine, Department of General Internal Medicine, Department of Emergency Intensive Care Unit, The First Affiliated Hospital of Sun Yat-sen University, No.58, Zhongshan 2nd Road, 510080 Guangzhou, China; 2 Department of Gastroenterology, The Third Affiliated Hospital of Sun Yat-Sen University, No.600, Tianhe Road, 510360 Guangzhou, China; 3 Department of Gastrointestinal Surgery, The First Affiliated Hospital of Sun Yat-sen University, No.58, Zhongshan 2nd Road, 510080 Guangzhou, China; 4 Department of Critical Care Medicine, The Sixth Affiliated Hospital of Sun Yat-sen University, No.26, YuanCunErHeng Road, 510655 Guangzhou, China

**Keywords:** Bacterial infection, Cell signalling

## Abstract

Intestinal mucosal integrity dysfunction during endotoxemia can contribute to translocation of intestinal bacteria and a persistent systemic inflammatory response, which both fuel the pathophysiological development of sepsis or endotoxemia. The pathogenesis of intestinal damage induced by endotoxemia remains poorly understood. Here, we identified the microRNA (miR)-674-5p/X-box binding protein 1 (XBP-1) axis as a critical regulator and therapeutic target in preventing intestinal crypt cell proliferation during endotoxemia. MiR-674-5p was markedly increased in intestinal epithelial cells (IECs) during endotoxemia and its induction depended on hypoxia-inducible factor-1α (HIF-1α). Intriguingly, gene expression microanalysis revealed that expression of XBP-1 was down-regulated in IECs with over-expression of miR-674-5p. miR-674-5p was found to directly target XBP-1 protein expression. Upon in vitro, anti-miR-674-5p enhanced sXBP-1 expression and facilitated intestinal crypt cell proliferation. Blockade of miR-674-5p promoted XBP-1 activity, attenuated intestinal inflammation, and expedited intestinal regeneration, resulting in protection against endotoxemia-induced intestinal injury in mice. More importantly, the survival in endotoxemia mice was significantly improved by inhibiting intestinal miR-674-5p. Collectively, these data indicate that control of a novel miR-674-5p/XBP-1 signaling axis may mitigate endotoxemia -induced intestinal injury.

## Introduction

Endotoxemia is the most common cause of mortality in most intensive care units and accounts for more than 250,000 deaths in the United States annually^1^. Endotoxemia is the host inflammatory response to severe, life-threatening infection, and results in organ dysfunction, including lung, kidney, and intestine^2^. Endotoxemia-induced intestinal injury is believed to have an important impact on the pathophysiology of endotoxemia and is considered the “motor” of the systemic inflammatory response^3,4^. Endotoxemia induces several aberrations in the intestinal epithelium involving barrier dysfunction^3,4^, magnified epithelial apoptosis^5–7^, and production of several inflammatory factors^8,9^. Moreover, intestinal epithelial integrity plays a vital role in physical barrier dysfunction induced by endotoxemia. The small intestinal epithelium normally renews every three and a half days with proliferated and differentiated cells moving from the crypts to the tip of villi^10^. Intestinal integrity, which is achieved by a balance of cell proliferation and cell death, has been shown to be injured in inflammatory pathological conditions such as endotoxemia or inflammatory bowel disease^11,12^. Recent evidence has suggested that impaired cell proliferation is a critical factor in disturbing intestinal epithelial integrity in endotoxemia^13^. Given that intestinal cell proliferation is important in endotoxemia-induced intestinal injury, researchers have been seeking protective agents for the intestine that would encourage intestinal cell proliferation and maintain intestinal cell homeostasis.

MicroRNAs (miRNAs) are a class of noncoding RNA molecules and endogenously expressed RNAs of 21–23 nucleotides that bind with partial sequence homology to the 3′-untranslated region (UTR) of target mRNAs and inhibit translation^14^. High-throughput and functional studies have shown that miRNAs play crucial roles in many aspects of cellular physiology as well as pathological processes such as inflammation and tumorigenesis^14,15^. Recently, miRNAs have been shown to function as modulators in the regulation of various aspects of gut epithelial homeostasis, including intestinal cell proliferation, apoptosis, and differentiation^16–18^. Several intestinal epithelial-specific miRNAs, including miR-222^17^, miR-322/503^19,20^, miR-21/155^21^, miR-195^22^, miR-122b^23^, and miR-29b^24^, have been found to modulate intestinal epithelial cell (IEC) proliferation, apoptosis, and cell-to-cell interaction. However, the roles of miRNAs in endotoxemia-induced intestinal injury remain to be explored.

In the present study, we identified changes in the expression of 10 miRNAs from a total of 30 novel miRNAs chosen from the miRNA expression profile of mouse embryos^25^ with endotoxemia-induced intestinal injury. Moreover, we identified miR-674-5p as a key miRNA that was markedly induced in endotoxemia-induced intestinal injury and was found to target X-box binding protein 1 (XBP-1), which in turn, inhibited intestinal crypt cell proliferation and exacerbated intestinal injury during endotoxemia.

## Results

### Upregulation of miR-674-5p in mouse IECs during endotoxemia-induced intestinal injury

**Fig. 1:**
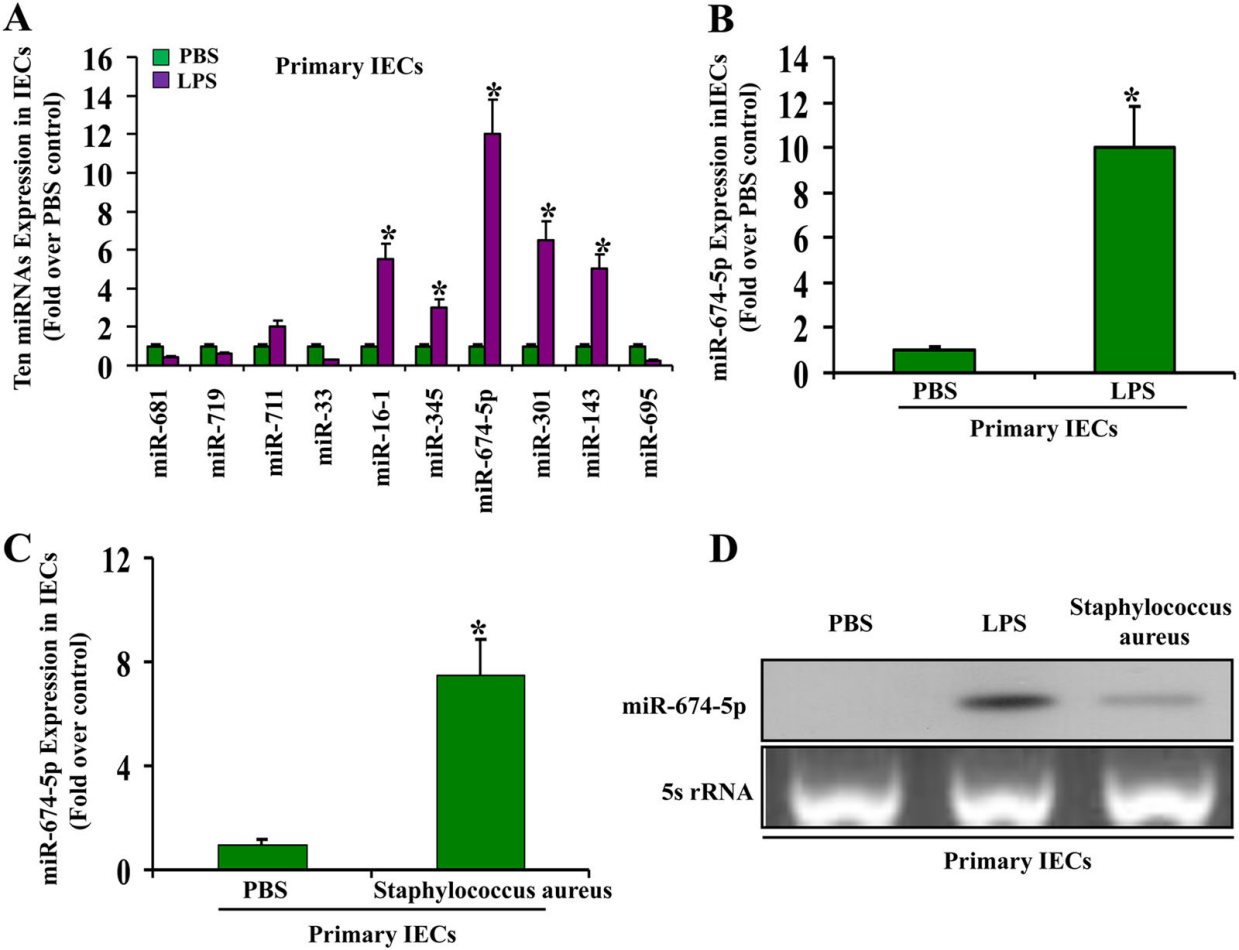
Upregulation of miR-674-5p in mouse IECs during endotoxemia-induced intestinal injury. **a** Significant changes in miRNA expression in IECs of small intestines isolated from mice at day 3 after treated with lipopolysaccharide (LPS) or phosphate-buffered saline (PBS) during endotoxemia-induced intestinal injury. Values are presented as means ± standard deviation (SD), *n* = 6 in each group. *P < 0.05 versus PBS. **b** Real-time PCR of miR-674-5p. RNA from IECs isolated from small intestines of mice at day 3 after injected with LPS or PBS. Values are presented as means ± SD, *n* = 6 in each group. *P < 0.01 versus PBS. **c** Real-time PCR of miR-674-5p. RNA from IECs isolated from small intestines of mice at day 3 after treated with *Staphylococcus aureus* or PBS. Values are presented as means ± SD, *n* = 6 in each group. *P < 0.05 versus PBS. **d** Northern blot analysis of miR-674-5p. Total RNA (10 μg) extracted from IECs isolated from mice at day 3 after treated with LPS, *S*. *aureus*, or PBS were used for northern blotting. 5S rRNA was probed as a loading control.

Total RNA was extracted from IECs isolated from small intestines of mice treated with LPS and used to investigate altered expression of miRNAs by real-time PCR. Of ~30 miRNAs selected from the expression profile of miRNAs in mouse embryos^25^, only 10 miRNAs in IECs of mice exhibited a significant change in expression following LPS treatment. While miRNAs 681, 719, 33, and 695 were downregulated, miRNAs 711, 16–1, 345, 674-5p, 301, and 143 were upregulated (Fig. 1a, b). Among these miRNAs, miR-674-5p exhibited the highest upregulation following LPS treatment. A similar change in miR-674-5p expression was also observed in IECs of endotoxemia mice treated with *S*. *aureus* (Fig. 1c). miR-674-5p induction in IECs during endotoxemia-induced intestinal injury was confirmed by northern blotting (Fig. 1d). miR-674-5p was previously identified via parallel signature sequencing technology, but its targets and function have remained elusive. Next, we investigated the function and significance of miR-674-5p in mouse IECs following LPS treatment.

### HIF-1α mediates induction of miR-674-5p during LPS stimulation

**Fig. 2:**
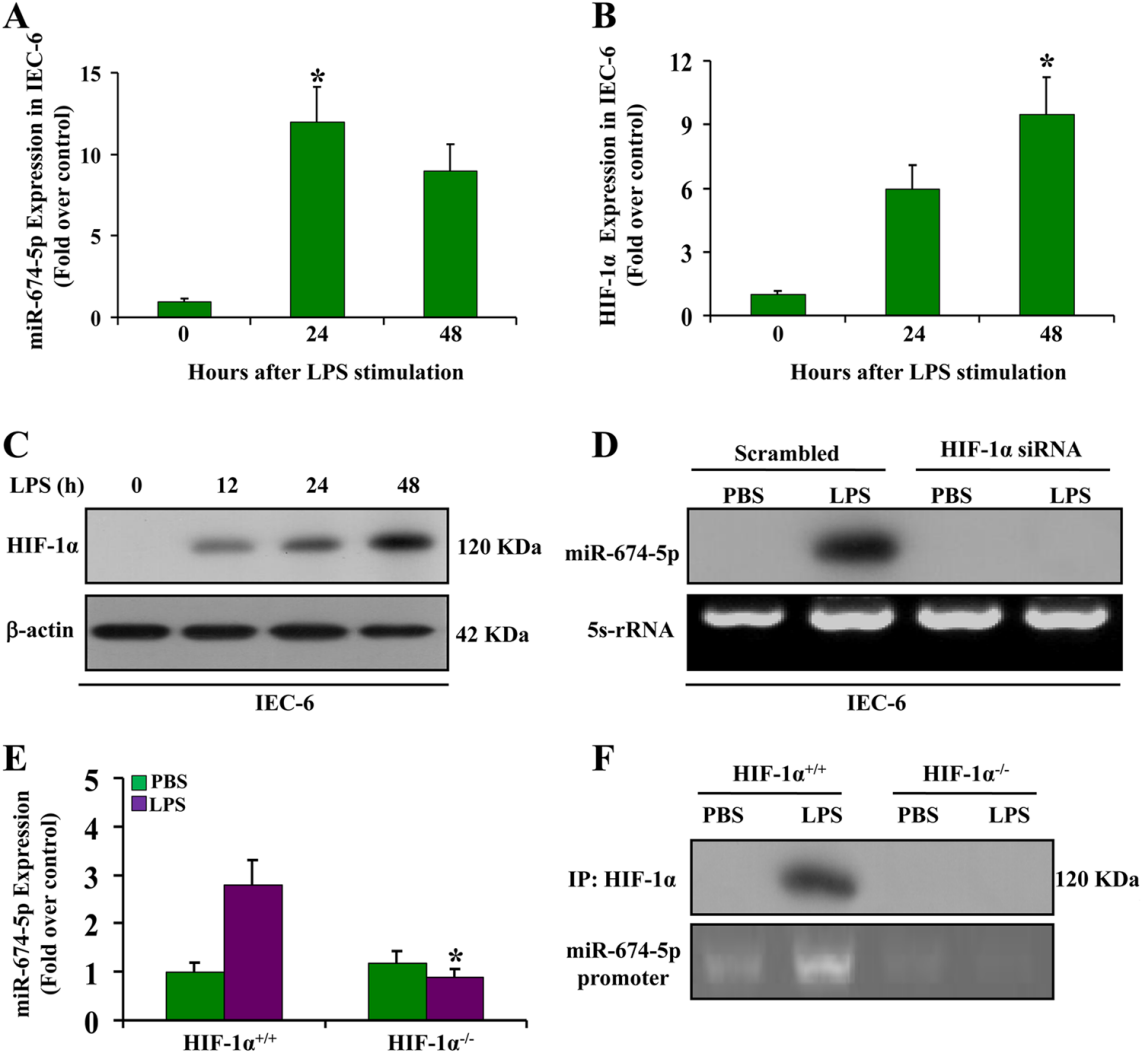
HIF-1α mediates induction of miR-674-5p during LPS stimulation. **a** Induction of miR-674-5p by LPS stimulation. IEC-6 cells were stimulated for 12–48 h to extract RNA for real-time PCR analysis of miR-674-5p. Fold changes over the value of IECs treated with PBS (arbitrarily set as 1) are shown. Values are presented as means ± SD, *n* = 6 in each group. *P < 0.01 versus IECs treated with PBS. **b** Induction of HIF-1α by LPS stimulation. IEC-6 cells were stimulated for 12–48 h to extract RNA for real-time PCR analysis of HIF-1α. Fold changes over the value of IEC cells treated with PBS (arbitrarily set as 1). Values are presented as means ± SD, *n* = 6 in each group. *P < 0.05 versus IECs with PBS. **c** Western blot analysis of HIF-1α in IEC-6 cells was carried out with whole cell lysates collected at various time points after LPS stimulation. **d** IEC-6 cells transfected with a sequence-scrambled oligonucleotide control or HIF-1α small interfering RNA were subjected to LPS stimulation, and whole cell lysates were collected at 24 h. **e** Induction of miR-674-5p by LPS stimulation. HIF-1α^+/+^ and HIF-1α^–/–^ mouse embryonic fibroblasts were stimulated for 24 h to extract RNA for real-time PCR analysis of miR-674-5p. Fold changes over the value of HIF-1α^+/+^ cells with LPS (arbitrarily set as 1) are shown. *P < 0.01 versus HIF-1α^+/+^ cells with LPS. **f** HIF-1α binding to the miR-674-5p promoter during LPS stimulation. HIF-1α^+/+^ and HIF-1α^–/–^ cells were stimulated with LPS or PBS for 24 h. Cell lysates were collected for chromatin immunoprecipitation analysis of HIF-1α binding to miR-674-5p promoter DNA.

HIF-1α has been shown to be critical in regulating inflammatory gene expression in various tissues and organs, including the intestine^26–28^. Therefore, we explored the connection between HIF-1α and miR-674-5p induction during LPS stimulation. In vitro, LPS stimulation markedly induced miR-674-5p and HIF-1α in IEC-6 cells (Fig. 2a, b). Moreover, HIF-1α was gradually increased at 48 h after LPS stimulation (Fig. 2c). Following HIF-1α knockdown which was confirmed by HIF-1α siRNA, miR-674-5p induction was significantly reduced (Fig. 2d). To further investigate whether miR-674-5p induction depends on HIF-1α, wild-type and HIF-1α-null MEFs were stimulated with LPS and miR-674-5p expression was analyzed. We found that miR-674-5p was promoted by LPS stimulation in wild-type MEFs but not in HIF-1α-null MEFs (Fig. 2e). Additionally, ChIP analysis demonstrated the binding of HIF-1α to the promoter region of miR-674-5p in MEFs (Fig. 2f). These results suggest that HIF-1α is important in inducing miR-674 expression under LPS stimulation.

### Expression of XBP-1 is decreased in miR-674-5p-treated human IECs

**Fig. 3:**
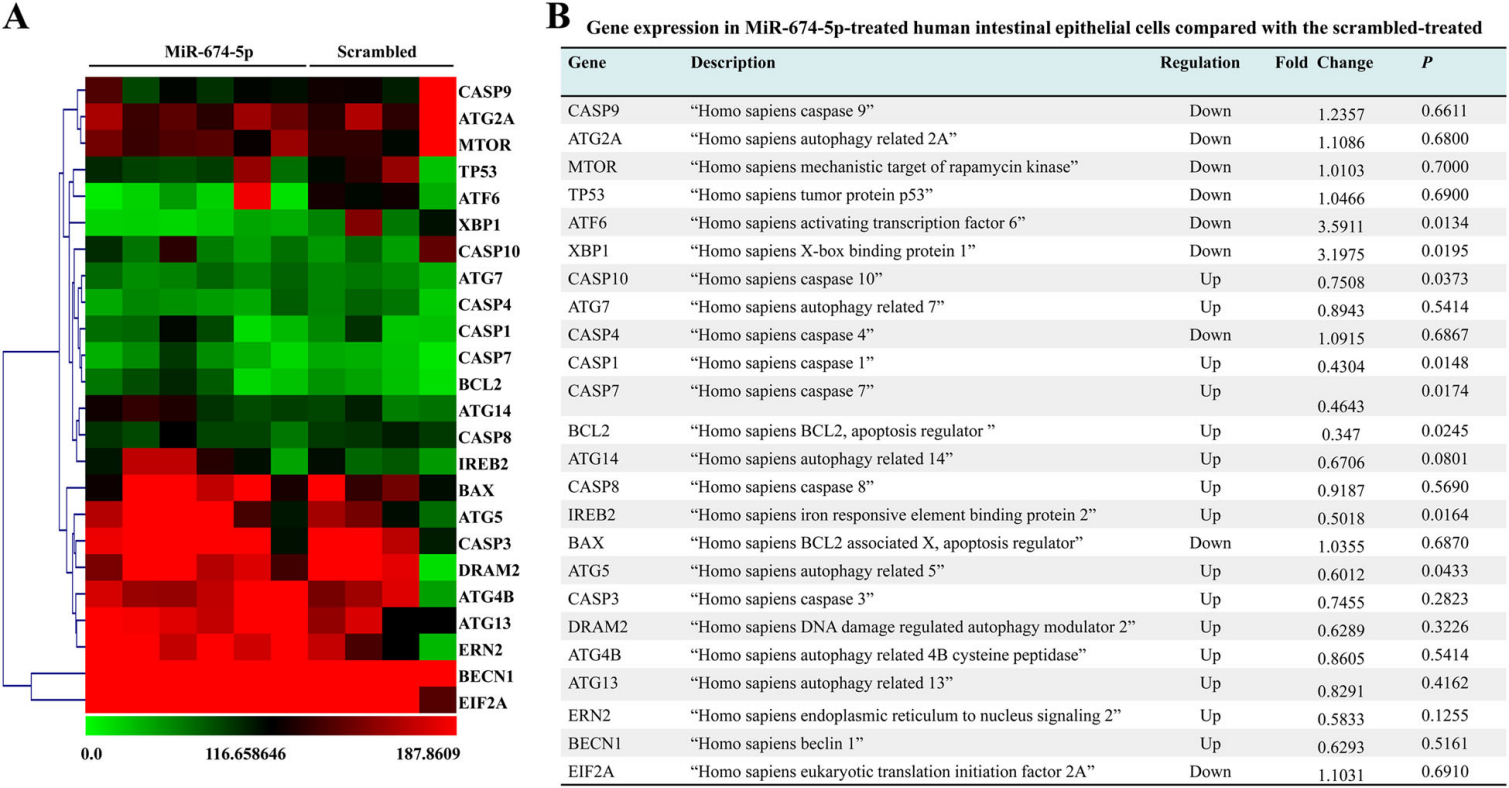
Expression of XBP-1 is decreased in miR-674-5p-treated human IECs. **a** Two-dimensional hierarchical clustering for apoptosis-, autophagy-, and endoplasmic reticulum (ER) stress-related genes among human IECs-FHC cells in 24 h after transfected with miR-674-5p or scrambled treatment. Fold changes in mRNA levels in FHC cells treated with miR-674-5p or scrambled are represented by green and red squares, showing decreased and increased levels, respectively. **b** Apoptosis-, autophagy-, and ER stress-related genes among human IECs-FHC cells in 24 hours after transfected with with miR-674-5p or scrambled treatment. The ratio represents the expression value in FHC cells with miR-674-5p-treated FHC cells compared with scramble-treated FHC cells.

Gene expression microanalysis was performed to screen for changes in gene expression of several apoptosis-, autophagy-, and endoplasmic reticulum (ER) stress-related genes in human intestinal epithelial cells (FHCs) treated with miR-674-5p or the scrambled. We found that expression of CASP10, CASP1, CASP7, BCL-2, IREB2, and ATG5 was significantly increased by less than one-fold (Fig. 3a, b). On the contrary, only two genes, ATF-6 and XBP-1, showed a significant decrease in expression of more than three-fold in miR-674-5p-treated FHCs (Fig. 3a, b). This indicates that miR-674-5p may regulate XBP-1 expression.

### XBP-1 over-expression improves IEC proliferation under LPS stimulation

**Fig. 4:**
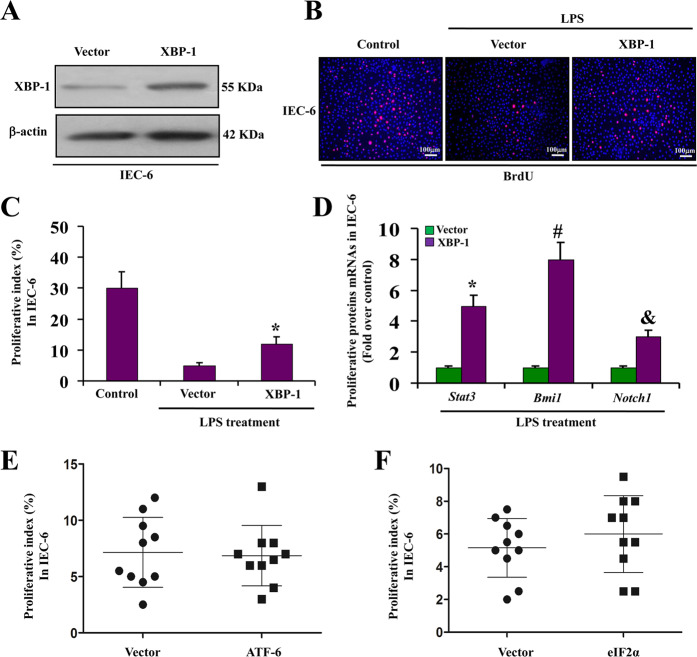
XBP-1 overexpression improves IEC proliferation under LPS stimulation. **a** Western blot analysis of XBP-1 in IEC-6 cells at 24 h with or without transfection of XBP-1. β-actin was used as a loading control. **b** BrdU staining of IEC-6 cells at 24 h after LPS stimulation with or without transfection of XBP-1. **c** Proliferative index was measured at 24 h after LPS stimulation by counting a minimum of 20 randomly selected microscopy fields (×200 magnification) following BrdU staining. The index was obtained by dividing the number of BrdU-positive cells by the total number of cells. *P < 0.01 versus vector control. **d** Real-time PCR analysis of STAT3, Bmi1, and Notch1. RNA from IEC-6 cells treated with LPS. *P < 0.05 versus vector control, ^#^*P* < 0.01 versus vector control, and ^&^*P* < 0.05 versus vector control. **e** Proliferative index was measured at 24 h after LPS stimulation with transfection of ATF-6 by counting a minimum of 20 randomly selected microscopy fields (×200) following BrdU staining. The index was obtained by dividing the number of BrdU-positive cells by the total number of cells. *P < 0.05 versus vector control. **f** Proliferative index was measured at 24 h after LPS stimulation with transfection of eIF-2α by counting a minimum of 20 randomly selected microscopy fields (×200) following BrdU staining. The index was obtained by dividing the number of BrdU-positive cells by the total number of cells. *P < 0.01 versus vector control.

Gene expression microanalysis demonstrated that expression of XBP-1 and ATF-6 is important in LPS-treated IECs. Since XBP-1 has previously been shown to facilitate cell survival and increase epithelial neoplasia in several pathological conditions^29,30^, we investigated the role of XBP-1 in endotoxemia-induced IEC injury. Following in vitro transfection of XBP-1 (Fig. 4a, b), cell proliferation was greatly enhanced in IEC-6 cells overexpressing XBP-1 when subjected to LPS stimulation (Fig. 4b, c). XBP-1 transfection upregulated the expression of XBP-1 and other proliferative proteins such as STAT3, Bmi1, and Notch1 (Fig. 4a, d). Increased cell proliferation was not observed in IEC-6 cells with ATF-6 or eIF-2α transfection following LPS treatment (Fig. 4e, f). These data confirm that XBP-1 has a protective impact on LPS-induced IEC injury in vitro.

### miR-674-5p targets XBP-1 during LPS stimulation

**Fig. 5:**
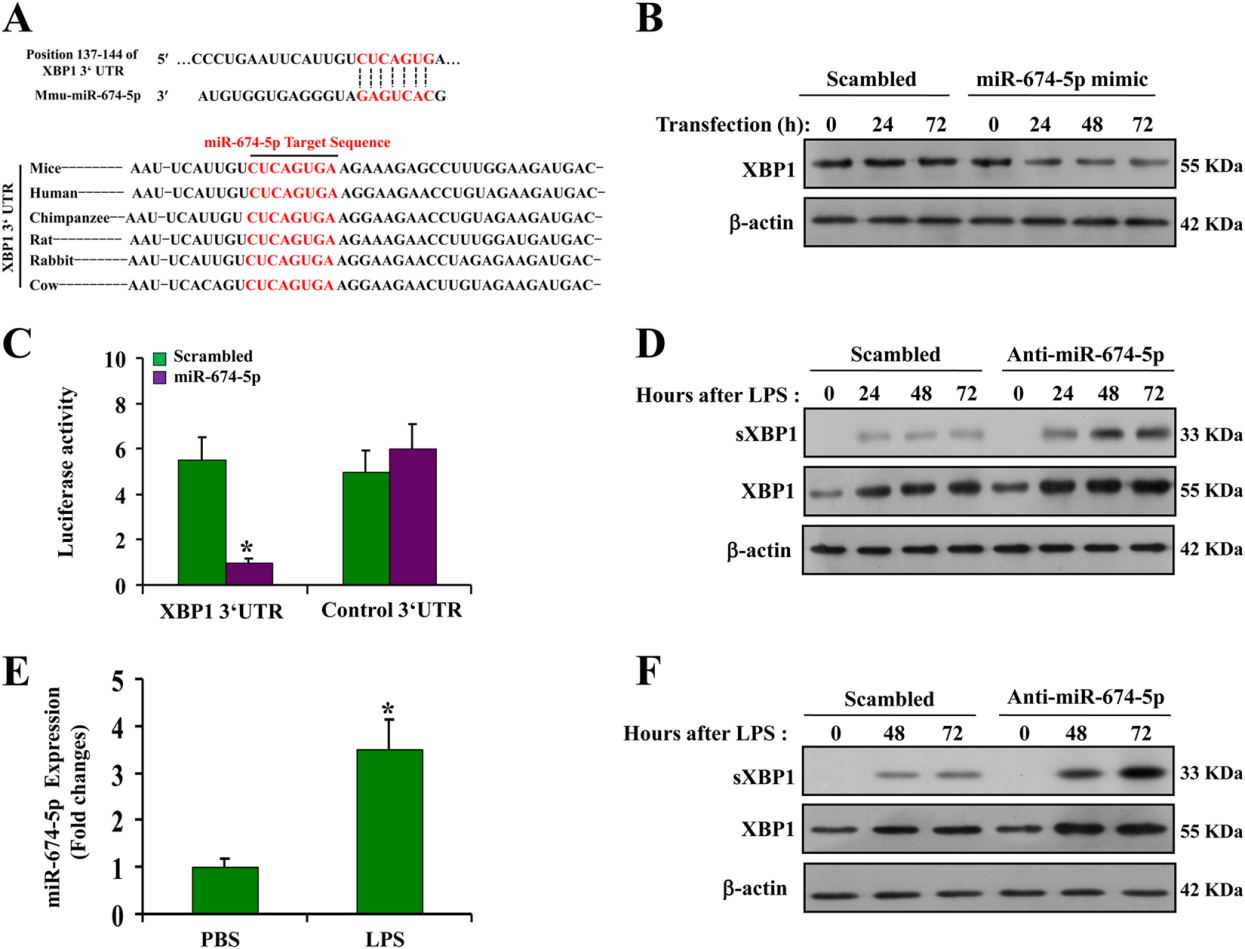
miR-674-5p targets XBP-1 during LPS stimulation. **a** Upper: putative miR-674-5p complementary sequence in the 3′ untranslated region (3′ UTR) of murine XBP-1 mRNA. Lower: conserved miR-674 target sequence in the XBP-1 3′ UTR. **b** CCC-HIE-2 cells were transfected with a sequence-scrambled oligonucleotide control or miR-674-5p and western blotting was carried out from whole cell lysates collected at various time points. **c** Luciferase reporter assay was conducted using constructs with the XBP-1 3′ UTR or an antisense control sequence. CCC-HIE-2 cells were cotransfected with these constructs along with the scrambled miRNA or miR-674 mimic. *P < 0.05 versus control 3′ UTR. Three independent experiments were performed. **d** IEC-6 cells transfected with a scrambled control or anti-miR-674 oligonucleotide were stimulated with LPS, and whole cell lysates were collected at the indicated time points. Three independent experiments were performed. **e** Quantitative PCR analysis of miR-674-5p gene expression at 24 h in CT-26 cells treated with LPS. *P < 0.05 versus PBS. **f** CT-26 cells transfected with a scrambled control or anti-miR-674 oligonucleotide were stimulated with LPS, and whole cell lysates were collected at the indicated time points. Western blotting of these lysates is shown.

Next, we investigated whether XBP-1 is a downstream target of miR-674-5p. Using the Targetscan database, target prediction indicated that XBP-1 may be a potential target of miR-674-5p. miR-674-5p was found to bind to the 3′ UTR of XBP-1 mRNA in various animal species (Fig. 5a). Transfection of a miR-674-5p mimic decreased XBP-1 expression in CCC-HIE-2 cells after 72 h (Fig. 5b). Furthermore, we explored the effect of miR-674-5p on the 3′ UTR of XBP-1 using the miRNA target luciferase reporter system. The 3′ UTR of XBP-1 and its reverse sequence (control 3′ UTR) were cloned downstream of the luciferase reporter gene activated by a constitutive promoter. The constructs were transfected into CCC-HIE-2 cells with miR-674-5p mimic or a sequence-scrambled oligonucleotide. The miR-674-5p mimic significantly weakened luciferase expression in luciferase-XBP-1 3′ UTR-transfected cells, while the sequence-scrambled oligonucleotide had no effect (Fig. 5c). As expected, luciferase expression was not significantly changed with the miR-674-5p mimic or the sequence-scrambled oligonucleotide in luciferase-control 3′ UTR-transfected cells. Our results suggest that miR-674-5p directly blocks XBP-1 expression. In IEC-6 cells, XBP-1 expression remained relatively stable following LPS stimulation, but a dramatic increase in sXBP-1 and XBP-1 expression was observed following treatment with the anti-miR-674-5p oligonucleotide (Fig. 5d). Similarly, in mouse colonic adenocarcinoma CT-26 cells, miR-674-5p expression increased following LPS stimulation and blocking miR-674-5p markedly upregulated sXBP-1 and XBP-1 expression (Fig. 5e). These results indicate that miR-674-5p directly targets XBP-1 expression under conditions of LPS stimulation.

### Suppression of miR-674-5p protects against endotoxemia-induced intestinal injury by regulating XBP-1

**Fig. 6:**
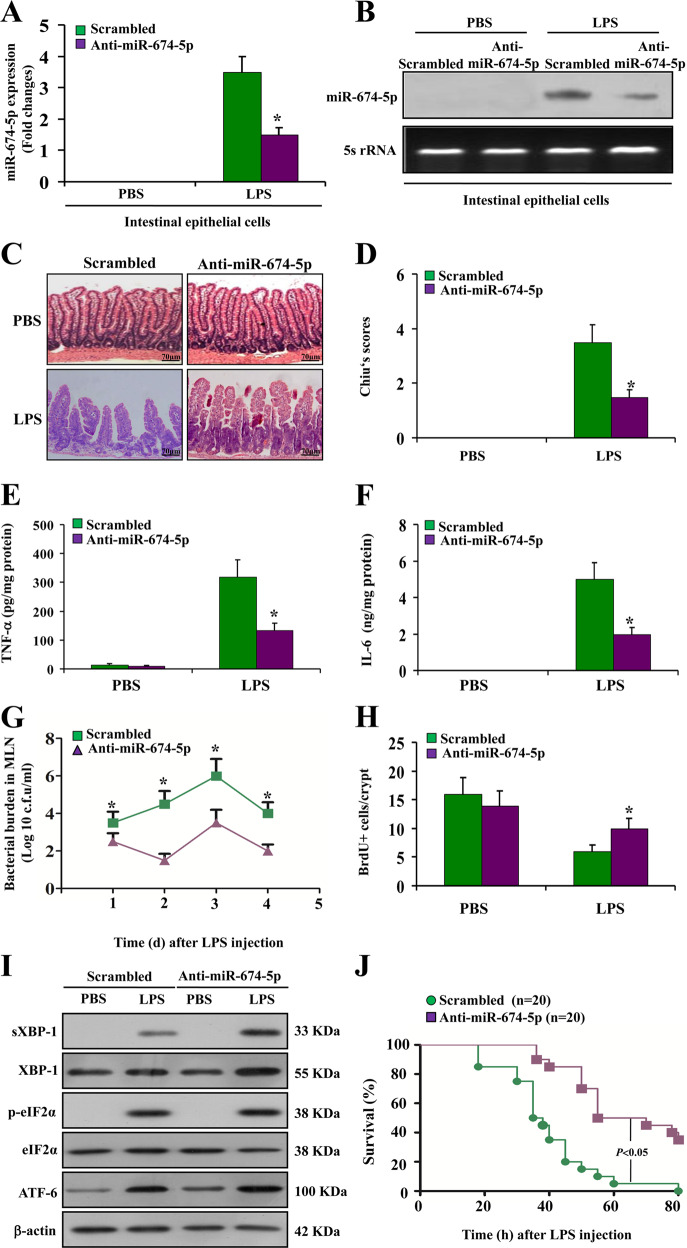
Suppression of miR-674-5p protects against endotoxemia-induced intestinal injury by regulating XBP-1. **a** Real-time PCR of miR-674-5p. RNA from IECs at 72 h after LPS treatment in mice with or without treatment with anti-miR-674-5p oligonucleotide. Values are presented as means ± SD, *n* = 6 in each group. *P < 0.01 versus PBS. **b** Northern blot analysis of miR-674-5p. Total RNA (10 μg) extracted from IECs isolated from mice at 72 h after LPS treatment was used for northern blotting. 5S rRNA was probed as a loading control. **c** Hematoxylin and eosin staining was performed using formalin-fixed tissue sections at day 5 after LPS treatment in mice with or without treatment with anti-miR-674 oligonucleotide or the sequence-scrambled oligonucleotide control. Magnification, ×400. **d** Chiu’s scores were measured and compared by analysis of variance with Tukey’s post-hoc test. *P < 0.01 versus scrambled control. Values are presented as means ± SD, *n* = 6 in each group. **e** Levels of TNF-α were measured at 72 h after LPS treatment in small intestinal mucosa of endotoxemia mice with or without treatment with anti-miR-674 oligonucleotide or the scrambled control. *P < 0.05 versus scrambled. Values are presented as means ± SD, *n* = 6 in each group. **f** ELISA analysis of IL-6 protein expression at 72 h after LPS treatment in small intestinal mucosa of endotoxemia mice with or without treatment with anti-miR-674 oligonucleotide or the scrambled control. *P < 0.01 versus scrambled. Values are presented as means ± SD, *n* = 6 in each group. **g** Bacterial counts in mesenteric lymph nodes at continuous time points after LPS treatment in endotoxemia mice with or without treatment with anti-miR-674 oligonucleotide or the scrambled control. *P < 0.05 versus scrambled. Values are presented as means ± SD, *n* = 6 in each group. **h** Average number of BrdU-positive cells in each crypt at 72 h following LPS treatment was determined by counting BrdU-positive cells in intact crypts. Values are presented as means ± SD, *n* = 6 in each group. *P < 0.05 versus scrambled control. **i** Western blot analysis of ER stress-related proteins of IECs isolated from endotoxemia mice with or without treatment with anti-miR-674 oligonucleotide or the scrambled control. β-actin was used as a loading control. **j** Survival curves of endotoxemia mice with or without treatment with anti-miR-674 or the scrambled control.

To study the role of miR-674-5p in endotoxemia-induced intestinal injury in vivo, we used systemic injection of anti-miR-674-5p oligonucleotide to specifically lower miR-674-5p expression in mouse IECs. With injection of anti-miR-674-5p oligonucleotide three times, miR-674-5p was significantly downregulated in IECs (Fig. 6a, b). Mice that received anti-miR-674-5p oligonucleotide showed significantly less intestinal epithelium injury than those that received the sequence-scrambled oligonucleotide control (Fig. 6c, d). The inflammatory biomarkers Tumor necrosis factor (TNF)-α and interleukin (IL)-6 were also reduced in small intestinal mucosa of anti-miR-674-5p-treated mice compared the control (Fig. 6e, f). Given that intestinal inflammation is closely associated with intestinal permeability and integrity, a bacterial burden assay was performed and intestinal proliferation was assessed. We found that the bacterial burden was markedly alleviated (Fig. 6g) and IEC proliferation was significantly improved in endotoxemia mice with anti-miR-674-5p oligonucleotide (Fig. 6h). Further analysis revealed that with LPS-induced ER stress, blocking miR-674-5p distinctly boosted XBP-1 expression, but not that of eIF-2α and ATF-6 (Fig. 6i). XBP-1 has been shown to promote cell survival in pathophysiological conditions^29^. The morphological and molecular changes in endotoxemia mice treated with anti-miR-674-5p oligonucleotide suggest that inhibition of miR-674-5p prolongs survival of endotoxemia mice (Fig. 6j). These results suggest that miR-674-5p-mediated downregulation of XBP-1 is important for the development of endotoxemia-induced intestinal injury.

### Blockade of miR-674-5p encourages intestinal crypt cell proliferation via XBP-1 under LPS stimulation

**Fig. 7:**
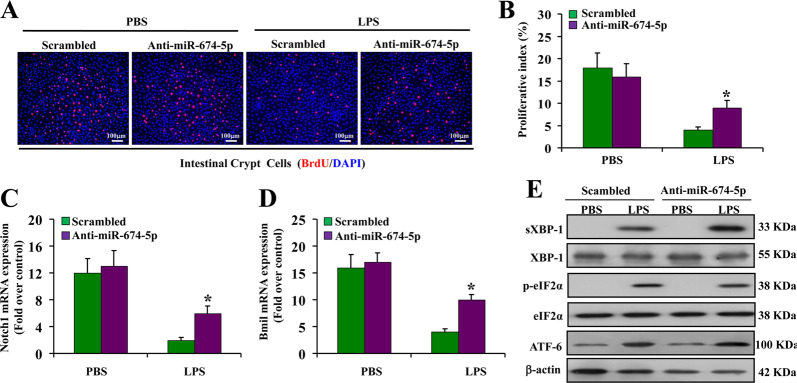
Blockade of miR-674-5p encourages intestinal crypt cell proliferation via XBP-1 under LPS stimulation. **a** BrdU staining of intestinal crypt cells isolated from mice at 24 h after LPS stimulation with or without treatment with anti-miR-674 oligonucleotide or the sequence-scrambled oligonucleotide control. **b** Proliferative index was measured at 24 h after LPS treatment by counting a minimum of 20 randomly selected microscopy fields (×200 magnification) following BrdU staining. The index was obtained by dividing the number of BrdU-positive cells by the total number of cells. *P < 0.01 versus scrambled control. **c** RT-PCR analysis of Notch1 mRNA in intestinal crypt cells isolated from mice at 24 h after LPS treatment with or without treatment with anti-miR-674 oligonucleotide or the scrambled control. *P < 0.05 versus scrambled control. **d** RT-PCR analysis of Bmi1 mRNA in intestinal crypt cells isolated from mice at 24 h after LPS treatment with or without treatment with anti-miR-674 or the scrambled. *P < 0.01 versus scrambled control. **e** Western blot analysis of ER stress-related proteins of intestinal crypt cells at 24 h after LPS stimulation with or without treatment with anti-miR-674 oligonucleotide or the scrambled control. β-actin was used as a loading control.

The above in vivo experiment implied that blocking miR-674-5p has a protective effect in endotoxemia-induced intestinal injury via regulation of XBP-1. We further studied the effect of miR-674-5p in vitro under LPS stimulation. Intestinal crypt cells, regarded as intestinal progenitor/stem cells, were isolated from small intestine of mice. Ki67, known as an frequently-used measurement for propagation, was solely expressed in intestinal crypts, not in villi and in isolated crypt cells (Supplementary Fig. 1A–C). Isolated crypt cells could form large colonies within 21 days (Supplementary Fig. 1D). However, proliferative capacity of intestinal crypt cells was reduced by 75% following LPS treatment, and was subsequently increased by ~50% following treatment with anti-miR-674-5p oligonucleotide (Fig. 7a, b). The two biomarkers for proliferation Notch1 and Bmil were greatly down-regulated in isolated intestinal crypt cells after LPS treatment, which could be significantly promoted by anti-miR-674-5p (Fig. 7c, d). More importantly, the ER stress-related proteins sXBP-1, but not ATF-6 and eIF-2α, were significantly increased in intestinal crypt cells treated with anti-miR-674-5p oligonucleotide compared with the sequence-scrambled oligonucleotide control (Fig. 7e). These results further support that miR-674-5p inhibits proliferation of intestinal crypt cells in response to LPS treatment through XBP-1 pathway.

## Discussion

This study identified an miRNA-mediated signaling pathway that regulates endotoxemia-induced intestinal injury. We demonstrated that miR-674-5p is a critical mediator in preventing IEC proliferation in the intestine in response to endotoxemia. Tissue microarray analysis and the luciferase assay demonstrated that XBP-1 was a direct target of miR-674-5p and that the miR-674-5p-mediated decrease in XBP-1 increased intestinal inflammation and inhibited intestinal crypt cell proliferation. Inhibiting miR-674-5p markedly mitigated intestinal injury induced by endotoxemia and increased survival. To our knowledge, there are only a few studies in which a single miRNA has been reported to significantly exacerbate intestinal epithelial damage following endotoxemia or sepsis by inhibiting IEC proliferation.

To study the role of miR-674-5p in endotoxemia-induced intestinal injury in vivo, systemic injection of anti-miR-674-5p oligonucleotides to block miR-674-5p in IECs was performed. miR-674-5p was found to markedly inhibit IEC proliferation under conditions of endotoxemia, suggesting that directly blocking miR-674-5p expression in IECs may be a potential therapeutic target to alleviate intestinal injury following endotoxemia. Moreover, miR-674-5p induction in IECs could be directly controlled by modulating HIF-1α expression. Several studies have indicated that HIF-1α expression is significantly induced under inflammatory conditions^26–28,31–33^, which is consistent with our results (Fig. 2). HIF-1α-mediated changes in miRNA expression were found to have a critical effect on the initiation and development of several pathophysiological processes, including ischemic kidney injury, colitis, and gastric cancer^26,34–37^. The HIF-1α/miRNA pathway induced by inflammation may be a universal feature of inflammation-associated diseases. In this study, induction of HIF-1α following LPS stimulation in IECs facilitated the increase in miR-674-5p expression, resulting in cell proliferation impairment. This was supported by the evidence that the expression of miR-674-5p was abrogated in the absence of HIF-1α in LPS-treated cells (Fig. 2), suggesting that control of the HIF-1α/miR-674-5p pathway exerts cyto-protective effects in endotoxemia-induced intestinal injury. However, whether miR-674-5p modulates HIF-1α in intestinal injury caused by endotoxemia remains to be answered.

XBP-1 is a member of the CREB/ATF basic region-leucine zipper family of transcription factors and functions as a key factor in the unfolded protein or ER stress response^38^. As one of three mechanistically distinct arms of the ER stress response, which include the Ire1α/XBP-1, PERK/eIF2α, and ATF-6 pathways, cleavage of cytoplasmic XBP-1 by the endoribonuclease Ire1α under conditions of ER stress results in nuclear translocation and upregulation of its target genes, the protein products of which operate in ER-associated degradation, the entry of proteins into the ER, and protein folding, which ultimately regulate inflammation, the immune system, and cell proliferation^39–49^. Previous studies have demonstrated that XBP-1 can modulate cell proliferation and tissue regeneration. In angiogenesis, XBP-1 was found to boost vascular endothelial cell proliferation via growth factor signaling pathways. XBP-1 was also shown to be crucial for smooth muscle cell proliferation through transforming growth factor (TGF)-β-mediated pathways that accelerate neointimal formation^42,43^. Moreover, in epithelial cell homeostasis, XBP-1 appears to be required for proliferation of pancreatic acinar cells, β-cells, and hepatocytes, which in turn, expedite pancreatic and liver regeneration^44,46^. In epithelial malignant neoplasms such as esophageal squamous cell carcinoma and breast cancer, XBP-1 could promote malignant cell propagation via different signaling pathways^47,48^. In this study, we demonstrated that blocking XBP-1 decreased the proliferation of IECs during endotoxemia and that by restraining miR-674-5p expression, enhanced XBP-1 could accelerate IEC proliferation, especially in the crypts, in endotoxemia-induced intestinal injury. However, one study showed that XBP-1 deficiency in IECs resulted in epithelial hyperproliferation via activation of STAT3 signaling^49^. The disparity between our findings and this study might be partly interpreted by the fact that PERK and XBP-1 act as two important branches of the ER stress response that can both promote IEC proliferation. IECs with knockout of XBP-1 exhibited unresolved ER stress due to hyperactivation of Ire1α; pathological ER stress could result in high expression of PERK, which could encourage IEC proliferation following injury^49^. In this study, blocking XBP-1, but not the PERK pathway, could mitigate IEC proliferation. Moreover, we identified the targeting of XBP-1 by miR-674-5p as a potential therapeutic target for improving endotoxemia-induced intestinal injury.

In conclusion, this study has highlighted miR-674-5p as a critical miRNA in alleviating endotoxemia-induced intestinal injury. Elaboration of the HIF-1α/miR-674-5p/XBP-1 signaling pathway not only provides novel and important insight into the pathogenesis of intestinal injury by endotoxemia or endotoxemia, but also suggests a novel miRNA-based therapeutic target for prevention and treatment.

## Materials and methods

### Animals, experimental sepsis and endotoxemia induction, and anti-miRNAs

The current study was approved by the Animal Care and Use Committee of Sun Yat-sen University, Guangzhou, China (approval number: 2018007). Experimental endotoxemia and sepsis model was induced respectively by administering lipopolysaccharide (LPS) from *Escherichia coli* (17.5 mg/kg, O55:B5; Sigma-Aldrich, St. Louis, MO, USA) intraperitoneally at a dose of 350 μg in 100 μL of saline or *Staphylococcus aureus* (10^8^ colony forming units [CFU] per mouse; ATCC 14458, SEB^+^TSST-1^–^) intravenously to 4–6-week-old mice weighing ~20 g. C57BL/6 male mice were monitored at 4-h intervals through critical stages of disease and euthanized with chloral hydrate at objective, predefined endpoints: loss of circulation to tail or feet, loss of responsiveness to stimuli, or breathing rate <120 breaths per minute. Survivors were monitored intensively for 6 days and euthanized 15 days after injection of LPS. Small intestines were harvested 3 days after injection of LPS for immunological, histopathological, serological, and bacteriological analyses.

Anti-miRNA administration was performed as described elsewhere^50^. Separate solutions of anti-miR-674-5p oligonucleotide and its scrambled negative control (Ambion, Austin, TX, USA) were diluted with in vivo-jetPEI solution (Polyplus-transfection) containing 10% (wt/vol) glucose at a ratio of in vivo-jetPEI nitrogen residues per oligonucleotide phosphate of 5, according to the manufacturer’s instructions. All solutions were shaken for 10 s and incubated for at least 15 min at 37 °C prior to injection. Each mouse received 400 μL of saline and oligonucleotide mixture (corresponding to 300 μg of oligonucleotide per dose) through tail vein injection consecutively for at least 3 days before experimental endotoxemia, and continuously received it until tissue collection or for at most 6 days after LPS injection. The intestines were harvested 24 h after the last injection. All injections were performed using a 30-gauge needle syringe with a single mouse restrainer.

### Histology and intestinal BrdU staining

A segment of the small intestine was stained with hematoxylin and eosin. Damage of the intestinal mucosa was evaluated using the criteria of Chiu’s method^51^ by two independent experienced pathologists who were blinded to the study groups. A minimum of six randomly chosen fields of view from each mouse were evaluated under a microscope and averaged to determine mucosal damage, and the results of the two pathologists were averaged.

Mice were injected with BrdU (150 mg/kg; Sigma-Aldrich) 4 h prior to sacrifice. For BrdU staining, sections were deparaffinized and treated with proteinase K (20 μg/mL) for 20 min at 37 °C. The staining was performed following a standard protocol with anti-BrdU antibody (1:100 in 5% bovine serum albumin [BSA], Sigma-Aldrich) and secondary antibody (Santa Cruz Biotechnology, Inc., Santa Cruz, CA, USA), and color was developed using a DAB kit (DaKo, Copenhagen, Sweden). BrdU-positive cells were counted in high-magnification (×400) fields, and the percentage of BrdU-positive cells in total crypts was scored by counting 100 intact crypts as described in the proliferative index and reported as the mean ± standard deviation (SD). Eight mice were evaluated in each group.

### Isolation of intestinal crypt cells

Intestinal crypt cells were isolated and cultured as described in our previous study^52^. Briefly, isolated small intestines were opened longitudinally, and washed with cold phosphate-buffered saline (PBS). The tissue was chopped into ~5-mm pieces, and washed again with cold PBS. The tissue fragments were incubated in 2 mM EDTA with PBS for 30 min on ice. Following removal of the EDTA medium, the tissue fragments were vigorously suspended using a 10-ml pipette with cold PBS. This fraction was passed through a 70-mm cell strainer (BD Biosciences, Franklin Lakes, NJ, USA) to remove residual villous material. Isolated crypts were centrifuged at 150–200×*g* for 3 min to separate crypts from single cells. The final crypts were counted and pelleted. A total of 500 crypts were mixed with 50 μl of Matrigel (BD Bioscience) and plated in 24-well plates. After polymerization of Matrigel, 500 μl of crypt culture medium (DMEM/F12(Invitrogen)) containing growth factors (10–50 ng/ml EGF (Peprotech)), 500 ng/ml R-spondin 1 and 100 ng/ml Noggin (Peprotech)) was added. Isolated crypts were incubated in culture medium for 45 min at 37 °C, following by trituration with a glass pipette. Crypt cells were passed through cell strainer with a pore size of 20 μm and collected in culture medium.

### Total RNA extraction and real-time PCR

Total RNA was extracted from IECs isolated from small intestines of mice using the RNAgents Total RNA Isolation System (Promega, Madison, WI, USA) according to the manufacturer’s instructions. A total of 40 ng of RNA was reverse-transcribed into cDNA using the miRNA Reverse Transcription kit (Applied Biosystems, Foster City, CA, USA). The TaqMan MicroRNA Assay (Qiagen, Hilden, Germany) was used for real-time PCR using sequence-specific primers for cDNA synthesis and Taqman probes for real-time PCR. The expression of miRNAs was normalized to that of the rnu19 gene. Thirty novel miRNAs chosen from the miRNA expression profile of mouse embryos^25^ were analyzed. Their sequences are described in Table 1. The all primer sequences of forward miRNAs, shared reverse miRNAs, and mRNAs were purchased from Guangzhou RiboBio Co.; Ltd (13–14/F, Innovation Building C3, 182 Kexue Avenue, Science Park, Guangzhou 510663, China). The primer sequences of ten changed miRNAs were followed as below: miRNA-681: “CAGCCTCGCTGGCAGGCAGCT”, miRNA-719: Forward “ATCTCGGCTACAGAAAAATGTT”, miRNA-711: Forward “GGGACCCGGGGAGAGATGTAAG”, miRNA-33: “GTGCATTGTAGTTGCATTGCA”, miRNA-16-1: “TAGCAGCACGTAAATATTGGCG”, miRNA-345: “GCTGACCCCTAGTCCAGTGCTT”, miRNA-674-5p: “GCACTGAGATGGGAGTGGTGTA”, miRNA-301: “GCTCTGACTTTATTGCACTACT”, miRNA-143: “GGTGCAGTGCTGCATCTCTGG”, miRNA-695: “AGATTGGGCATAGGTGACTGAA”. HIF-1α: Forward “TCTCGGCGAAGCAAAGAGTC”, Reverse “AGCCATCTAGGGCTTTCAGATAA”.

**Table 1:** Thirty miRNAs and their sequences.

Name	Sequence
mmu-miR-181b-1	AACAUUCAUUGCUGUCGGUGGGU
mmu-miR-423	AGCUCGGUCUGAGGCCCCUCAGU
mmu-miR-337	UCAGCUCCUAUAUGAUGCCUUU
mmu-miR-370	GCCUGCUGGGGUGGAACCUGGU
mmu-miR-379	UGGUAGACUAUGGAACGUAGG
mmu-miR-412	UUCACCUGGUCCACUAGCCG
mmu-miR-376b	AUCAUAGAGGAACAUCCACUU
mmu-miR-153	UUGCAUAGUCACAAAAGUGAUC
mmu-miR-681	CAGCCUCGCUGGCAGGCAGCU
mmu-miR-27b	UUCACAGUGGCUAAGUUCUGC
mmu-miR-195	UAGCAGCACAGAAAUAUUGGC
mmu-miR-711	GGGACCCGGGGAGAGAUGUAAG
mmu-miR-719	AUCUCGGCUACAGAAAAAUGUU
mmu-miR-16-1	CCAGUAUUGACUGUGCUGCUGA
mmu-miR-15a	UAGCAGCACAUAAUGGUUUGUG
mmu-miR-33	GUGCAUUGUAGUUGCAUUGCA
mmu-miR-615	UCCGAGCCUGGGUCUCCCUCUU
mmu-miR-688	UCGCAGGCGACUACUUAUUC
mmu-miR-690	AAAGGCUAGGCUCACAACCAAA
mmu-miR-691	AUUCCUGAAGAGAGGCAGAAAA
mmu-miR-693	CAGCCACAUCCGAAAGUUUUC
mmu-miR-301	CAGUGCAAUAGUAUUGUCAAAG
mmu-miR-133a-3	UUUGGUCCCCUUCAACCAGCUG
mmu-miR-345	GCUGACCCCUAGUCCAGUGCUU
mmu-miR-145	GUCCAGUUUUCCCAGGAAUCCCU
mmu-miR-143	UGAGAUGAAGCACUGUAGCUC
mmu-miR-146b	UGAGAACUGAAUUCCAUAGGCU
mmu-miR-669b	AGUUUUGUGUGCAUGUGCAUGU
mmu-miR-695	AGAUUGGGCAUAGGUGACUGAA
mmu-miR-674-5p	GCACUGAGAUGGGAGUGGUGUA

The English in this document has been checked by at least two professional editors, both native speakers of English. For a certificate, please see: http://www.textcheck.com/certificate/TnOlxO.

### Cell culture, treatments, and proliferative index

The human embryonic intestinal mucosa-derived cell line CCC-HIE-2, Hypoxia inducible factor (HIF)-1α^+/+^ and HIF-1α^–/–^ mouse embryonic fibroblasts (MEFs) were originally obtained from the National Infrastructure of Cell Line Resource (Chinese Academy of Medical Sciences). The rat small intestine cell line IEC-6 was obtained from the American Type Culture Collection (ATCC; Manassas, VA, USA). CCC-HIE-2 and IEC-6 cells were cultured in complete medium consisting of Dulbecco’s modified Eagle medium (DMEM) or DMEM/F12 (Thermo Fisher Scientific, Waltham, MA, USA), respectively, supplemented with 10% fetal bovine serum at 37 °C in a CO_2_ incubator.

Anti-miR-674-5p oligonucleotide (100 nmol/L) and its scrambled negative control (Ambion) were used in in vitro experiments and transfected into CCC-HIE-2 cells, IEC-6 cells, or MEFs for 24 h prior to stimulation with LPS (100 ng/mL) for 24 h.

XBP-1, activating transcription factor (AFT)-6, or protein kinase R-like endoplasmic reticulum kinase (PERK; 100 nmol/L) (Thermo Fisher Scientific) or their scrambled negative controls were used in in vitro experiments and transfected into IEC-6 cells for 24 h prior to stimulation with LPS (100 ng/mL) for 24 h.

For BrdU staining, BrdU was co-cultured with CCC-HIE-2 or IEC-6 cells for 4 h before harvesting. Staining was performed according a standard protocol with anti-BrdU antibody (1:100 in 5% BSA; Sigma-Aldrich) and secondary antibody (Santa Cruz Biotechnology, Inc.), and color was developed using a DAB Kit (DaKo). The proliferative index was determined by dividing the number of BrdU-positive cells by the total number of cells in at least 20 randomly selected fields (×200 magnification). Three independent experiments were performed.

### Bacterial culturing

For CFU analysis of *E*. *coli* in mesenteric lymph nodes (MLNs), we harvested and homogenized MLNs in sterile PBS, which were serially diluted and plated followed by incubation at 37 °C for 24 h.

### Northern blot analysis

The sequences for probing miR-647-5p in northern blot were “TACACCACTCCCATCTCAGTGC” and internal control: “CACGGGAAGTCTGGGCTAAGAGACA”, which were purchased from Shanghai Generay Biotech Co., Ltd (13–14 Building, No.5398 Shenzhuan Rd, Songjiang District, Shanghai, 201619 China). Northern blot analysis of miRNAs was conducted according to the manufacturer’s instruction. Briefly, 10 μg of total RNA isolated using the Ambion RNA extraction kit (Applied Biosystems) was resolved using a 15% acrylamide-bisacrylamide gel (19:1) containing 7 M urea in Tris-borate-EDTA buffer. Following transfer to a Hybond membrane (Amersham, Uppsala, Sweden) and ultraviolet crosslinking, the membrane was incubated with the radiolabeled hybridization probe in Ultra-Hyb-oligo hybridization buffer (Ambion). The membrane was then washed extensively before exposure to X-ray film at –70 °C.

### Chromatin immunoprecipitation

Chromatin immunoprecipitation (ChIP) analysis of HIF-1α binding to the miR-674-5p promoter was performed using an assay kit (R&D Systems, Minneapolis, MN, USA) according to the manufacturer’s instructions. The primer sequences of the miR-674-5p promoter were: Forward “GCTACCACATTTCATCTGACTAGAG”. Reverse “AGCAAGCACTTGATTTCACATAAC”, which was purchased from Shanghai Generay Biotech Co., Ltd (13–14 Building, No.5398 Shenzhuan Rd, Songjiang District, Shanghai, 201619 China). Briefly, following fixation with formaldehyde, cell lysates were collected and sonicated to shear chromatin. The samples were then centrifuged to collect the supernatant for immunoprecipitation with anti-HIF-1α antibody. After several washes, the resulting immunoprecipitates were subjected to PCR analysis using specific primers.

### Microarray experiment

The mixture, containing Lipofectamine (Invitrogen, Carlsbad, CA, USA) and miR-674-5p or scambled dissolved in Optimal (Invitrogen), was added to cells at 80% confluence, and 24 h after interference, and harvested. PCR was used to test successful transfection into cells.

The Agilent SurePrint G3 Human Gene Expression 8×60 K Array was designed with eight identical arrays per slide, with each array containing probes interrogating 27,958 Entrez Gene RNAs. The array also contained 1280 Agilent control probes. The four scrambled samples were human IECs (FHC cells) tranfected with the scramble sequence. The six miR-674-5p samples were human IECs (FHC cells) tranfected with the miR-674-5p. Total RNA from each sample was isolated using TRIzol reagent according to the manufacturer’s instructions (Invitrogen, Carlsbad, CA, USA) and was further purified using the mirVana miRNA Isolation Kit (Ambion) according to the manufacturer’s instructions. The purity and concentration of RNA was tested with OD260/280 readings using a spectrophotometer (NanoDrop ND-1000). cDNA labeled with the fluorescent dye Cy3-dCTP was constructed by Eberwine’s linear RNA amplification method and subsequent enzymatic reaction. The procedure was optimized using the CapitalBio cRNA Amplification and Labeling Kit (CapitalBio, Beijing, China) for producing high yields of labeled cDNA. DNA polymerase and RNase H were employed to synthesize double-stranded cDNA (dsDNA) and the dsDNA products were purified using a PCR NucleoSpin Extract II Kit and eluted with 30 μL of elution buffer. The eluted dsDNA products were evaporated to 16 μL and subjected to in vitro transcription reactions of 40 μL at 37 °C for 14 h using a T7 Enzyme Mix. A Klenow enzyme labeling strategy was used following reverse transcription using CbcScriptII reverse transcriptase. Array hybridization was carried out in a hybridization oven (Agilent Technologies, Santa Clara, CA, USA) overnight at a rotation speed of 20 rpm at 42 °C and washed with two consecutive solutions. Data summarization, normalization, and quality control of the array data were performed using GeneSpring software V12 (Agilent Technologies). To screen the differentially expressed genes, threshold values of ≥1.5- and ≤1.5-fold change and a Benjamini-Hochberg corrected *P* value of 0.05 were used. The data were log2 transformed and median centered by genes using the Adjust Data function of CLUSTER 3.0 software and then further analyzed with hierarchical clustering with average linkage. Finally, tree visualization was presented using Java Treeview software (Stanford University School of Medicine, Stanford, CA, USA). Data from this study are available from the National Center for Biotechnology Information under GEO accession number GSE67764.

### Plasmid constructs and luciferase reporter assay

The human 3′-UTR of the *XBP-1* gene was amplified by PCR using the primers XBP-1-3′-UTR-Forward (5′-GTAAGCAACGGGAACA-3′) and XBP-1-3′-UTR-Reverse (5′-AAATGGAGAAAGCACCT-3′) and cloned into the *Xba*I/*Xba*I site of the pGL3 control vector (Promega) to generate the vector pGL3-XBP-1. For the luciferase reporter assay, CCC-HIE-2 cells were cultured in 96-well plates and transfected with pGL3-XBP-1 + miR-674-5p mimic, pGL3-XBP-1, pGL3-control + miR-674-5p mimic, or pGL3-control using Lipofectamine 2000 (Invitrogen). At 48 h after transfection, cells were collected, washed with PBS, and analyzed with a dual-luciferase reporter assay system (Promega) according to the manufacturer’s instructions with a Lumat LB 9507 luminometer (Berthold, Nashua, NH, USA).

### Tumor necrosis factor-α and interleukin-6 assays

The concentrations of TNF-α and IL-6 in small intestinal mucosa of mice were measured using a commercial kit (eBioscience, San Diego, CA, USA) according to the manufacturer’s instructions. After the stop solution was added, the plates were read at 450 nm (570 nm correction) on a MicroPlate Reader (BioTek, Seattle, WA, USA). The results are expressed as pg TNF-α/mg protein and ng IL-6/mg protein.

### Western blotting and antibodies

Total protein (50 μg) of small IECs was denatured in sample buffer containing sodium dodecyl sulfate (SDS) and β-mercaptoethanol, separated using 4–20% gradient SDS-polyacrylamide gel electrophoresis, and transferred onto nitrocellulose membranes. Nonspecific binding sites of the membranes were blocked using defatted milk protein. The relative amount of primary antibody was detected with peroxidase-conjugated secondary antibody. Densitometry was used to quantify protein abundance. Similar procedures were performed with antibodies against XBP-1, GRP78, and ATF-6 (Sigma-Aldrich); cleaved caspase-3 and cleaved caspase-12, sXBP-1 (Cell Signaling Technology, Danvers, MA, USA); and HIF-1α, eukaryotic initiation factor (eIF)-2α, phosphorylated eIF2α (p-eIF2α), Ki67 (Abcam, Cambridge, UK).

### Statistical analysis

All experiments were performed at least in triplicate. Data are expressed as mean ± SD. Six mice were used in each group. Randomization was used in each independent experiment. Statistical significance was analyzed with the one-way or two-way ANOVA test for gene and protein expression, comparing miRNAs expression, cellular proliferation, luciferase activity, inflammatory factors, BrdU positive counts, and positive rate differences between two groups. The survival data were analyzed by log-rank test using GraphPad Prism software. Differences were considered significant if the probability of the difference occurring by chance was <0.05 (*P* < 0.05).

## Supplementary information

Supplementary Figure 1
