# Supplementary figures and images for: MicroRNA-674-5p induced by HIF-1α targets XBP-1 in intestinal epithelial cell injury during endotoxemia
Source: Cell Death Discov. 2020 Jun 4;6:44. doi: 10.1038/s41420-020-0280-5 (PMC7272402; doi:10.1038/s41420-020-0280-5)

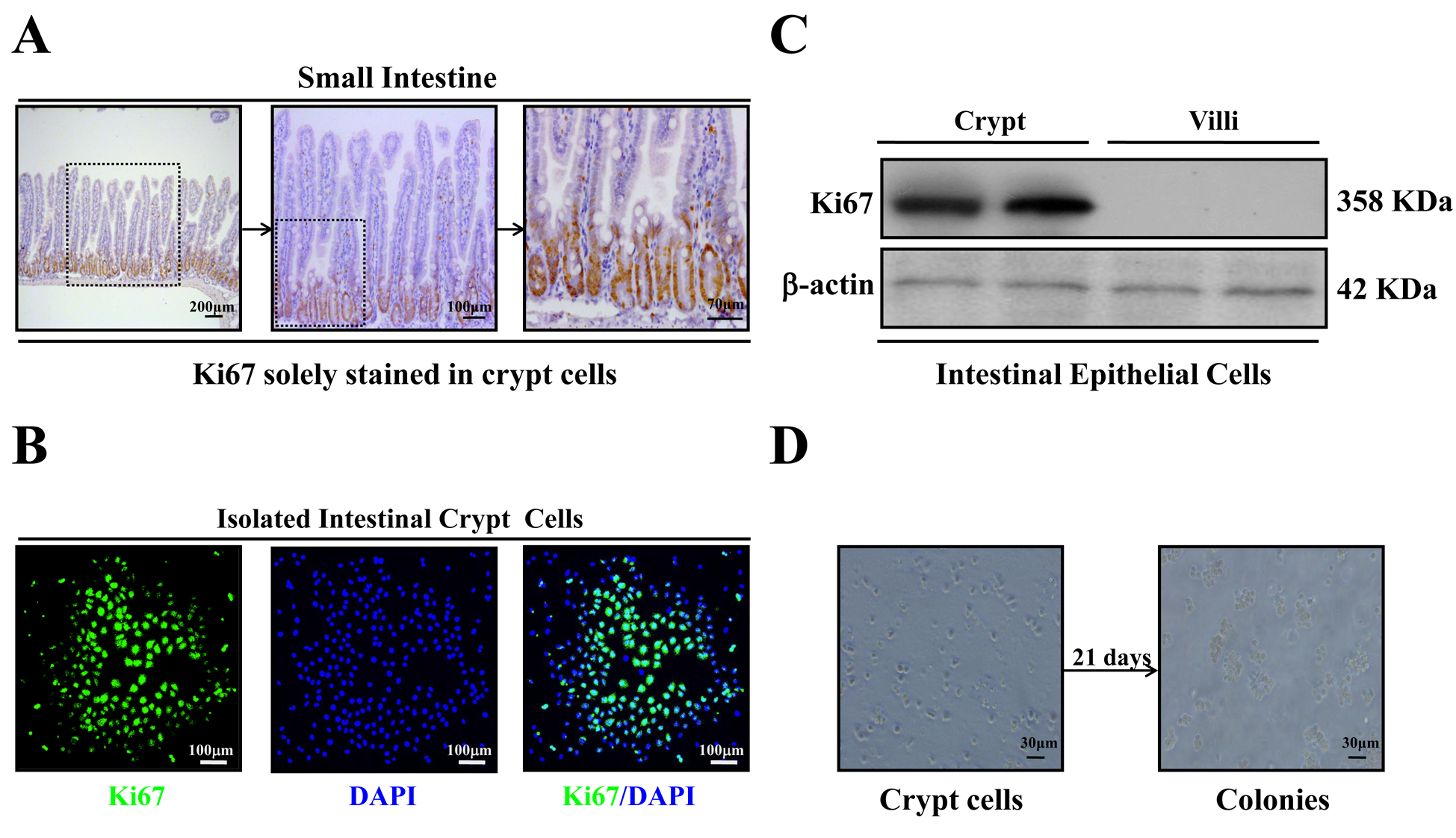

Supplement: Supplementary file 1 — Supplementary Figure 1 [file 41420_2020_280_MOESM1_ESM.tif]
